# Pathogens as Biological Weapons of Invasive Species

**DOI:** 10.1371/journal.ppat.1004714

**Published:** 2015-04-09

**Authors:** Andreas Vilcinskas

**Affiliations:** 1 Institute for Phytopathology and Applied Zoology, Justus-Liebig University of Giessen, Giessen, Germany; 2 Fraunhofer Institute for Molecular Biology and Applied Ecology, Department of Bioresources, Giessen, Germany; University of Wisconsin Medical School, UNITED STATES

## What Is the Role of Pathogens in the Spread of Invasive Species?

Invasive species are nonindigenous species that are introduced into new environments, where they become established and expand their range [1]. They undergo rapid proliferation following the colonization of new habitats, often at the expense of native species, thus having a negative impact on biodiversity. The prospering discipline of invasion ecology seeks to understand why some species become successful invaders, while others do not, even if they are closely related. Pathogens and parasites appear to play an important role in this context. The enemy release hypothesis proposes that invasive species are more competitive in newly colonized habitats because their natural enemies, including pathogens and parasites, are left behind in their native range [2]. Alternatively, the novel weapons hypothesis proposes that invasive species have acquired chemical weapons that provide a selective advantage over their competitors [3]. There is now convincing evidence that the novel weapons hypothesis can be expanded to include pathogens and parasites that are vectored by the invasive species into newly colonized habitats [1]. These pathogens and parasites can act as biological weapons if they infect and kill native competitors.

## Are Pathogens Often Used as Biological Weapons?

There are many examples of invasive species benefiting from their ability to carry pathogens or parasites that are harmless to the invasive host but lethal to indigenous species, therefore conferring a selective advantage on the invader even if the indigenous species is better adapted to the habitat. For example, native red squirrels (*Sciurus vulgaris*) have been replaced by the invasive gray squirrel (*S*. *carolinensis*) in the United Kingdom because the latter carry *Squirrel parapoxvirus*, which causes a fatal disease only in the red squirrel [4]. Similarly, the decline of the noble crayfish (*Astacus astacus*) in Europe has been caused by the fungal pathogen *Aphanomyces astaci*, which was cointroduced with the signal crayfish *Pacifastacus leniusculus* from North America [5]. In both examples, the invasive species has become more competitive by selectively weakening its opponent. A prerequisite for disease-mediated invasions is that the alien vector displays a higher tolerance than the indigenous species against the vectored pathogen or parasite. An intriguing example is the invasive ladybird *Harmonia axyridis*, which is native to central and eastern Asia but has been introduced to North America and Europe as a biological control agent against aphids and scale insects. This species, also known as the multicolored or harlequin ladybird, is now spreading around the world and has become a model in invasion biology because it can successfully outcompete native ladybird species in diverse new habitats. It has recently been shown to carry abundant microsporidia, which are fungi-related obligate parasites that replicate within eukaryotic cells after penetrating the plasma membrane using an extruded polar tube [6,7]. Microsporidia isolated from *H*. *axyridis* kill native ladybird species such as *Coccinella septempunctata* when experimentally transferred [8]. The tolerance of *H*. *axyridis* against its associated microsporidia may be mediated by the chemical defense compound harmonine, which displays activity against a wide range of bacteria and parasites [9].

## Does Innate Immunity Play a Role in Invasion Biology?

Species-dependent invasive success has been difficult to explain, but recent evidence suggests that it is more likely in animals with a better, more adaptable innate immune system. This allows naïve members of the species to survive initial encounters with novel pathogens to which they have not adapted by coevolution [10]. This invasive immunity hypothesis was recently supported by a comparative analysis of immune responses in *H*. *axyridis* and native ladybirds [11]. Next-generation sequencing of the immunity-related *H*. *axyridis* transcriptome identified more than 50 genes encoding putative antimicrobial peptides, the highest number reported thus far in any animal [12]. The remarkably large repertoire of antimicrobial peptides that can be induced by pathogen challenge, combined with the constitutive production of harmonine, explains how this species can be protected against pathogens and parasites it has never encountered and also how it maintains resistance against its incumbent microsporidial population [13]. The role of pathogens in the context of invasion biology therefore reflects several underlying mechanisms acting in concert.

## How Do Invaders Disseminate Their Biological Weapons?

**Fig 1:**
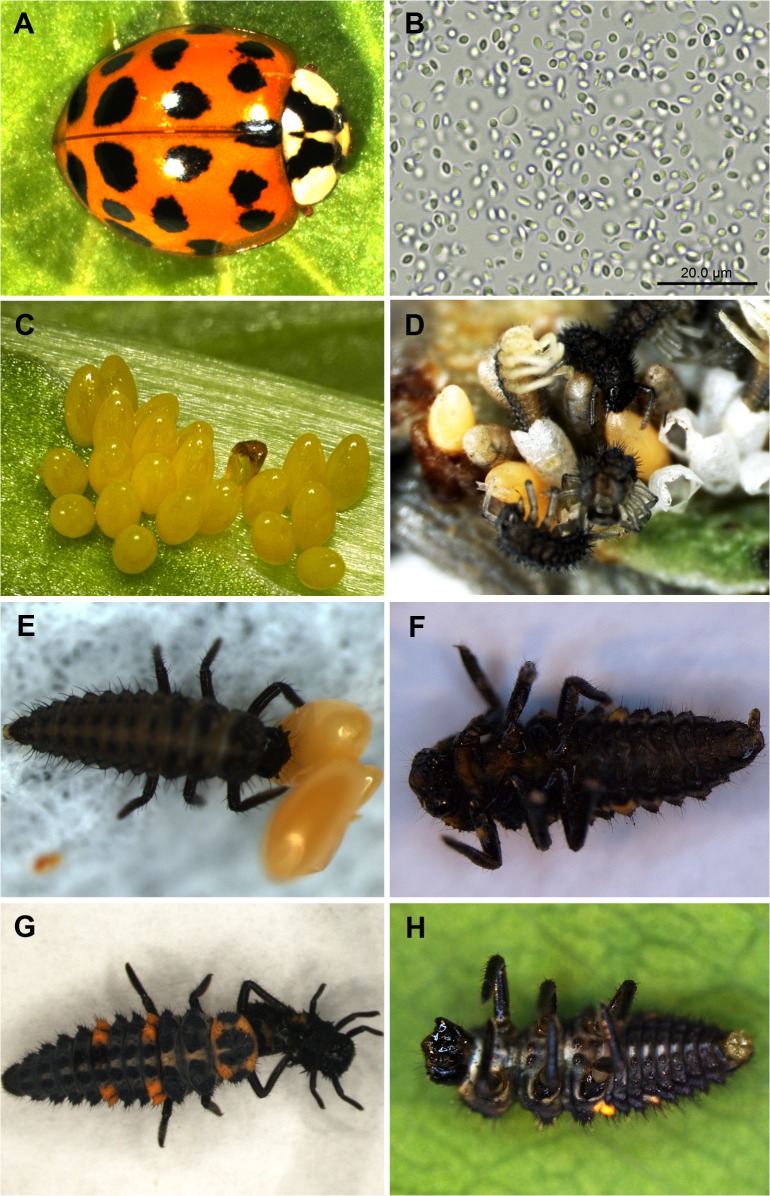
Transmission of pathogens from invasive to native ladybirds. The invasive ladybird *H*. *axyridis* (A) carries a high load of microsporidia in its hemolymph (B), and these parasites are vertically transmitted via the eggs (C) and larvae (D). Native predatory ladybird species such as the larvae of the two-spotted ladybird *Adalia bipunctata*, which feed *H*. *axyridis* eggs, can become infected via the oral uptake of microsporidia (E) and ultimately die (F). Similarly, larvae of the native seven-spotted ladybird *C*. *septempunctata* are known predators of smaller *H*. *axyridis* larvae (G). Intraguild predation has been postulated as a mechanism enabling the microsporidia carried by *H*. *axyridis* to cross the species barrier and infect native ladybirds (H).

The use of pathogens as biological weapons requires that the invasive species be able to disseminate them so that they are transferred to the competitor and can propagate in the indigenous population. For example, *Squirrel parapoxvirus* can only infect red squirrels when they are sympatric with the introduced North American gray squirrels, which represent a reservoir of the virus and contaminate the habitat with their urine [14]. Native crayfish in Europe have declined even in environments that have not yet been populated by introduced crayfish from Northern America, because the spores of the cointroduced fungal pathogen *A*. *astaci* can also be vectored by water birds and even humans who have visited waters with established populations of the invader [5]. A special transmission mechanism has been postulated for the microsporidia associated with *H*. *axyridis*. Microsporidial infection usually follows the oral uptake of spores [6,7]. In the case of ladybirds, microsporidia can cross the species barrier when predatory ladybirds feed on the contaminated eggs or larvae of other species (Fig. 1). The vertical transmission of microsporidia in *H*. *axyridis* has recently been documented [15]. Mutual cannibalism among ladybirds is known as intraguild predation and may therefore promote the dispersal of microsporidia introduced into the habitat by invasive ladybird species.

## Are Biological Weapons Always Lethal?

Pathogens and parasites cointroduced with invasive species are generally lethal to the native competitor in all the models studied thus far, but effective biological weapons do not need to kill the native species in order to benefit the invader. The balance could also be tipped in favor of the invader if the performance or fecundity of the native competitors is reduced, thus making the native species breed less quickly or compete less effectively for resources such as food. There is some evidence that microsporidia associated with invasive ladybirds reduce the number of offspring produced by native species following heterospecific transmission, resulting in a long-term population decline that favors the vectoring invader [15]. However, the relationship between invaders and native species is complex, and the use of biological weapons to reduce reproduction and fitness rather than to kill will need careful experimental evaluation, including field studies. It will be necessary to distinguish between the direct effect of pathogens and the indirect effect of the growth of the invasive population on the ability of native species to find food and reproduce successfully. Furthermore, we should take into account that cointroduced pathogens represent only one factor among many others that have been postulated in the literature to promote invasive success of invasive species such as *H*. *axyridis* [15,16].
